# LATENT CLASSES AND PROGRESSION OF MMSE IN YOUNG-ONSET DEMENTIA: DATA FROM THE SWEDISH DEMENTIA REGISTER

**DOI:** 10.1093/geroni/igae098.1723

**Published:** 2024-12-31

**Authors:** Deborah Finkel, Fanny Kårelind, Steven Zarit, Therése Bielsten, Helle Wijk, Linda Johansson

**Affiliations:** University of Southern California, Louisville, Kentucky, United States; Jönköping University, Jönköping, Jonkopings Lan, Sweden; The Pennsylvania State University, University Park, Pennsylvania, United States; Jönköping University, Jönköping, Jonkopings Lan, Sweden; Gothenburg University, Gothenburg, Ostergotlands Lan, Sweden; Institute of gerontology, School of Health and Welfare, Jönköping University, Jönköping, Sweden

## Abstract

Studies have shown significant heterogeneity in the longitudinal progression of dementia. Growth mixture models have detected up to 4 classes that differ in both baseline Mini-Mental State Exam (MMSE) and rate of decline over time. Most analyses focus on adults over age 65 and investigate group differences in demographic and health variables. The current analysis focused on adults with young onset dementia (YOD) and examined the role of demographic and support variables. Sample included 1025 adults (55% women) registered in the Swedish Dementia Register prior to age 65 with at least 3 registrations. Age at baseline was 38 to 64 (mean=59.3, SD=4.1); follow-up ranged from 1 to 12 years (mean=4.6, SD=2.0). Growth mixture models identified 4 classes: high baseline MMSE and moderate decline over time (56.1%), intermediate baseline MMSE and somewhat faster decline (39.2%), low baseline and stable MMSE over time (3.5%), and high baseline with steep decline (1.2%). Group 2 had the highest proportion Alzheimer’s Disease (AD) diagnosis and Group 4 had the lowest. Group 3 was more likely to have vascular or unspecified dementia and Group 4 was more likely to have vascular or frontotemporal dementia. Groups differed in age at diagnosis (Group 2 youngest), participation in adult daycare (Group 4 most likely), and having home health assistance (Group 2 most likely). Results highlight that YOD is just as heterogeneous as later onset dementia; therefore, it is vital that people with YOD get early diagnosis and a case manager to help identity and meet their individual needs.

